# Assessing the subcellular distribution of oncogenic phosphoinositide 3-kinase using microinjection into live cells

**DOI:** 10.1042/BSR20130133

**Published:** 2014-04-14

**Authors:** Meredith J. Layton, Natalie K. Rynkiewicz, Ivan Ivetac, Kristy A. Horan, Christina A. Mitchell, Wayne A. Phillips

**Affiliations:** *Department of Biochemistry and Molecular Biology, Monash University, VIC 3800, Australia; †Surgical Oncology Research Laboratory, Peter MacCallum Cancer Centre, Melbourne, VIC 3002, Australia; ‡Sir Peter MacCallum Department of Oncology, University of Melbourne, Parkville, VIC 3010, Australia; §Department of Surgery (St. Vincent's Hospital), University of Melbourne, Parkville, VIC 3010, Australia

**Keywords:** Cdc42, microinjection, mutation, oncogene, PI3K, *PIK3CA*, 2-ME, 2-mercaptoethanol, Akt, also called PKB (protein kinase B), BH, BCR-homology, cSH2, C-terminal SH2, DAPI, 4′,6-diamidino-2-phenylindole, DXMS, deuterium exchange mass spectrometry, EGF, epidermal growth factor, GAP, GTPase-activating protein, GFP, green fluorescent protein, GST, glutathione transferase, HRP, horseradish peroxidase, iMMEC, immortalized mouse mammary epithelial cells, iSH2, inter-SH2, MDCK, Madin–Darby canine kidney, NA, numerical aperture, nSH2, N-terminal SH2, PDGF, platelet-derived growth factor, PH, pleckstrin homology, PI, phosphatidylinositol, PI3K, phosphoinositide 3-kinase, PIP_2_, phosphoinositide-4,5-disphosphate, PIP_3_, phosphoinositide-3,4,5-trisphosphate, PM, plasma membrane, PS, phosphatidylserine, PTEN, phosphatase and tensin homologue deleted on chromosome 10, pY, phosphotyrosine, RTK, receptor tyrosine kinase, SH, Src homology

## Abstract

Oncogenic mutations in *PIK3CA* lead to an increase in intrinsic phosphoinositide kinase activity, but it is thought that increased access of PI3Kα (phosphoinositide 3-kinase α) to its PM (plasma membrane) localized substrate is also required for increased levels of downstream PIP_3_/Akt [phosphoinositide-3,4,5-trisphosphate/also called PKB (protein kinase B)] signalling. We have studied the subcellular localization of wild-type and the two most common oncogenic mutants of PI3Kα in cells maintained in growth media, and starved or stimulated cells using a novel method in which PI3Kα is pre-formed as a 1:1 p110α:p85α complex *in vitro* then introduced into live cells by microinjection. Oncogenic E545K and H1047R mutants did not constitutively interact with membrane lipids *in vitro* or in cells maintained in 10% (v/v) FBS. Following stimulation of RTKs (receptor tyrosine kinases), microinjected PI3Kα was recruited to the PM, but oncogenic forms of PI3Kα were not recruited to the PM to a greater extent and did not reside at the PM longer than the wild-type PI3Kα. Instead, the E545K mutant specifically bound activated Cdc42 *in vitro* and microinjection of E545K was associated with the formation of cellular protrusions, providing some preliminary evidence that changes in protein–protein interactions may play a role in the oncogenicity of the E545K mutant in addition to the well-known changes in lipid kinase activity.

## Abstract 2

Oncogenic forms of PI3Kα (phosphoinositide 3-kinase α) microinjected into live cells are not recruited to the PM (plasma membrane) to a greater extent, and do not reside at the PM longer, than wild-type PI3Kα.

## INTRODUCTION

The *PIK3CA* gene encodes the p110α subunit of the Class 1A PI3K (phosphoinositide 3-kinase). The prototypic Class 1A PI3K exists as a heterodimer of a catalytic p110α subunit and a regulatory p85α subunit (p110α/p85α or PI3Kα) [1,2] and phosphorylates the phosphoinositide lipid, PIP_2_ (phosphoinositide-4,5-disphosphate), at the 3′ position of the inositide ring to form PIP_3_ (phosphoinositide-3,4,5-trisphosphate) [3]. Somatic, mono-allelic, single base mutations in *PIK3CA* that result in single amino acid substitutions are found frequently in breast and colon cancers [4–7] and have been shown to be oncogenic [8–11].

The p110 and p85 subunits of PI3Kα contain several functional domains. p110α contains a p85α-binding domain, a Ras-binding domain, a C2 domain, a helical domain and a kinase domain. The p85α subunit contains an SH3 (Src homology 3) domain, a GAP (GTPase-activating protein)-like domain, an nSH2 (N-terminal SH2) domain, an iSH2 (inter-SH2) domain that binds p110α and a cSH2 (C-terminal SH2) domain. The most common oncogenic *PIK3CA* mutations are E545K in the p110α helical domain and H1047R in the p110α kinase domain [8,12]. These mutated forms of PI3Kα (p110α^E545K^/p85α and p110α^H1047R^/p85α) are associated with increased PIP_3_ levels [9,10,13,14] and up-regulation of Akt [also called PKB (protein kinase B)] signalling [9,15]. PI3K/PIP_3_ signalling regulates a wide range of fundamental cellular processes including cell proliferation, survival, glucose metabolism and cell migration [1–3].

PI3Kα is not an integral membrane protein and so must be recruited to the PM (plasma membrane) to gain access to its PM-localized substrate, PIP_2_. Binding to a number of PM-associated proteins, such as activated RTKs (receptor tyrosine kinases), activated Ras, SH3 domain-containing proteins and small GTPases, has been reported to activate PI3Kα [16–18]. However, the extent to which these interactions activate the intrinsic lipid kinase activity or activate PI3Kα by translocating it to the PM is not clear [19,20]. Some oncogenic mutations are thought to primarily up-regulate enzymatic activity. For example, p110α is both inhibited and structurally stabilized by tight binding to the p85α subunit [21] and it has been proposed that the intrinsic kinase activity of PI3Kα can be activated by disruption of an inhibitory contact between the p85α nSH2 domain and the p110α catalytic domain, which can occur due to the binding of the nSH2 and cSH2 domains to specific pY (phosphotyrosine)-containing motifs (pYXXM) present in RTKs [22–24] or due to the E545K mutation [18,25]. Other oncogenic mutations are proposed to primarily mediate an interaction with the PM [25,26]. For example, from the X-ray crystal structure of p110α^H1047R^ in complex with the iSH2 and nSH2 domains of p85α, it has been proposed that the p110α C2 domain, along with a region of the iSH2 domain, forms a positively charged contact surface for negatively charged membrane lipids [25,26] and that the H1047R mutation alters the conformation of 13 residues near the C-terminus of p110α to form a loop that cooperates with the C2 and iSH2 domains to mediate a constitutive interaction with the PM and thus increases lipid kinase activity by allowing easier access to PIP_2_ [25].

Although p110α^E545K^/p85α and p110α^H1047R^/p85α have been reported to bind lipids better than p110α^wt^/p85α *in vitro* [27], the subcellular localization of the wild-type and mutant PI3Kα, and their distribution between the cytosol and PM, has not been studied. Here, we have used a novel approach of microinjection of fluorescently labelled, highly purified, recombinant p110α/p85α complexes to quantify the degree of PM localization of wild-type and oncogenic mutant PI3Kα in cells maintained in growth media, and starved or stimulated cells. We found no difference in the interaction of the wild-type versus mutant PI3Kα with PM lipids *in vitro* or in its subcellular distribution in intact cells. Instead, we observed increased numbers of cell protrusions in cells microinjected with p110α^E545K^/p85α and a higher affinity binding of p110α^E545K^/p85α to activated Cdc42, providing some preliminary evidence that point mutations in *PIK3CA* may affect protein–protein interactions in addition to its enzymatic activity.

## MATERIALS AND METHODS

### Reagents

MDCK3 (Madin–Darby canine kidney 3) epithelial cells [28], a gift from Professor Anne Ridley, King's College, London, U.K., were grown in DMEM (Dulbecco's modified Eagle's medium) containing 10% (v/v) FBS. Caco2(C2BBe1) colon epithelial cells were obtained from A.T.C.C. (#CRL-2102) and were grown in RPMI supplemented with 10% FBS. iMMEC (immortalized mouse mammary epithelial cells) were prepared as previously described [29] and grown in DMEM (Dulbecco's modified Eagle's medium)/F-12 (Invitrogen) supplemented with 10% FBS, 1 μg/ml hydrocortisone (Sigma), 5 μg/ml insulin (Sigma) and 5 ng/ml human EGF (epidermal growth factor; BD Transduction Labs). Antibodies used were GST (glutathione transferase; #71–7500, Invitrogen), p85α (#610045; BD Transduction Labs) and E cadherin (#610181; BD Transduction Labs). HRP (horseradish peroxidase)-conjugated secondary antibodies used for immunoblotting were from Chemicon. DAPI (4′,6-diamidino-2-phenylindole) and CellLight™ PM-GFP (green fluorescent protein) BacMam 2.0 used for immunofluorescence were from Molecular Probes. Recombinant human EGF (BD Transduction Laboratories) and insulin (Sigma) used for stimulation were reconstituted as a 10 × stock in FBS (500 ng/ml and 50 μg/ml, respectively). Lipids were purchased from either Sigma [PS (phosphatidylserine) #P5660, PI (phosphatidylinositol) #P2517, PI-(4,5)-P_2_ #P9763, Folch Fraction IV #B1502 and mixed PIs #P6023] or Echelon Biosciences [PI-3-P #P-3016, PI-(3,4)-P_2_ #P-3416 and PI-(3,4,5)-P_3_ #P-3916].

### Generation of recombinant proteins

Wild-type or mutant p110αEE/p85α complexes were expressed in insect (Sf9) cells and purified as previously described [30]. Plasmids encoding GST fusions of the PH (pleckstrin homology) domain of Akt1 and Grp1 or a GFP fusion of the PH domain from Btk (courtesy of Professor Tamas Balla, National Institute of Child Health and Human Development, Bethesda, MD, U.S.A.), constitutively active (V12) forms of H-Ras, Rac1 and Cdc42 (courtesy of Professor Anne Ridley) or control GST alone (pGEX-4T1, GE Healthcare) were transformed into the BL21 (DE3) *Escherichia coli* strain and protein expression was induced with 0.5 mM IPTG (isopropyl β-D-thiogalactopyranoside) for 4 h at 37°C. GST fusion proteins were purified from clarified bacterial cell lysates using glutathione agarose affinity chromatography. The concentrations of purified, recombinant proteins were quantified by UV spectroscopy using a molar extinction coefficient calculated from their amino acid composition (ProtParam).

### Fluorescent labelling of purified recombinant proteins

Purified, recombinant wild-type or mutant p110αEE/p85α complexes were buffer exchanged into 20 mM Bicine pH 8.2, 150 mM NaCl containing 10 mM 2-ME (2-mercaptoethanol) using Sephadex G-25 (GE Life Sciences), then incubated with a 100-fold molar excess of the maleimide mono-reactive form of the fluorophore Cy3 (GE Healthcare #PA23031) for 60 min at room temperature (22°C). Unreacted dye was inactivated by the addition of Tris pH 8.0 to a final concentration of 100 mM. GST fusion proteins were buffer exchanged into PBS and labelled with Alexa488 (Molecular Probes #A20000) as per the manufacturer's instructions. Cy3-labelled p110αEE/p85α complexes and Alexa488-labelled GST-fusion proteins were separated from free fluorophore and buffer exchanged into microinjection buffer [20 mM Tris (pH 7.5), 100 mM KCl, 50 mM NaCl, 0.1% (v/v) 2-ME in 40% (v/v) glycerol] using Sephadex G-25 and concentrated using centrifugal filters (Amicon Ultra15 10000 NMWL, Millipore). The number of coupled fluorophore molecules per protein molecule was determined by measuring the ratio of the absorbances at 280 and 550 nm for Cy3 or 495 nm for Alexa488 using a scanning UV spectrophotometer and molar extinction coefficients calculated from the amino acid composition or specified by the manufacturer (150 000 M^−1^cm^−1^ for Cy3 and 73 000 M^−1^cm^−1^ for Alexa488).

### Gel electrophoresis and immunoblotting

Purified recombinant proteins were separated by SDS–PAGE using 10% Tris–glycine gels. Gels of fluorophore-labelled proteins were fixed in 20% (v/v) methanol, 7% (v/v) acetic acid then scanned using a Typhoon scanner (GE Life Sciences) using a 532 nm laser and emission filters for Cy3 and Alexa488. For immunoblots, SDS–PAGE separated proteins were transferred onto a PVDF membrane, blocked and probed with antibodies that recognize either GST or p85α [1/1000 in 20 mM Tris pH7.5, 150 mM NaCl [TBS (Tris-buffered saline)] containing 0.01% (v/v) Tween 20] followed by HRP-labelled secondary antibodies (1/20 000) and detection using chemiluminescence (Pierce). Scanned immunoblots were quantified using ImageQuant (GE Life Sciences) software.

### PI3K assays

PI3K assays were carried out as previously described [31–33] in TBS containing 5 mM 2-ME. Assays contained 2 mM MgCl_2_, 2 mM MnCl_2_, 0.2 mM ATP, 5–10 μCi [^32^P]γATP, 500 μg/ml of PI and 250 μg/ml of PS. Extracted phospholipids were separated by TLC in 65% (v/v) 1-propanol, 0.7 M acetic acid, 50 mM phosphoric acid, exposed to a phosphor screen (Molecular Dynamics) and analysed using ImageQuant software.

### Microinjection

Fluorescently labelled, purified recombinant proteins in microinjection buffer were microinjected into adherent cells with Femtotips II microcapilliaries using a Femtojet microinjector coupled to an Injectman micromanipulator (Eppendorf) essentially as described [34] and according to the manufacturer's instructions. Cells to be microinjected were grown in 6 mm diameter × 1 mm deep wells made using a CultureWell Gasket (#CW-8R-1.0, Grace Biolabs) on a 24×60 mm Nr 1.5 glass coverslip (Menzel) or 35 mm μ-dishes with low walls and an imprinted 500 μm grid (#80156, Ibidi). Cells were microinjected in a media containing 10% FBS with an injection time of 1 s and a pressure of between 100 and 250 hPa depending on cell type, then left to recover for 2 h at 37°C in the same media before fixing or starving and stimulating. Alternately, to measure the effect of stimulation, microinjected cells were starved for 4 h in media containing no FBS at 37°C then stimulated for 10 min with 10% FBS, 50 ng/ml EGF and 5 μg/ml insulin to activate RTKs. For measurement of PM localization, cells (≤10% confluent) were infected with the PM marker CellLight™ PM-GFP according to the manufacturer's instructions 3 days prior to microinjection.

### Immunofluorescence

Microinjected cells were fixed with 4% (v/v) formaldehyde in PBS for 10 min. Nuclei were visualized by staining with DAPI (50 μg/ml, 5 min). For images of fixed cells, cells on glass coverslips were mounted using DPX (Sigma) and confocal images were collected using a Leica SP5 confocal microscope equipped with a ×100 [NA (numerical aperture) 1.45] oil immersion lens using standard filter sets and laser lines for Alexa488, Cy3 and DAPI.

For 3D image analysis of fixed cells, unmounted cells in 35 mm μ-dishes were washed and stored in PBS then imaged using a Nikon C1 motorized confocal microscope equipped with a ×60 (NA 1.35) water immersion lens. Cells were microinjected with different fluorescently labelled, purified recombinant proteins at specific grid locations. Immunofluorescence derived from Cy3 and GFP was measured at pre-defined grid locations in 80 successive focal planes between the top and the bottom surface of the cell using standard filter sets and laser lines.

For 3D image analysis of live cells, microinjected cells that had been allowed to recover post-microinjection for 2 h in DME containing 10% FBS in 35 mm μ-dishes were washed and starved in phenol red-free DME with no FBS for 4 h then imaged using a Nikon C1 motorized confocal microscope as above except that 25 successive focal planes of cells microinjected with different proteins at pre-defined grid locations were imaged prior to stimulation and then at 5, 15, 25, 35, 45 and 65 min post-stimulation with 10% FBS, 50 ng/ml EGF and 5 μg/ml insulin. Confocal images were processed and displayed using ImageJ (v 1.43) Software. Fluorescence intensity along a line was calculated and graphed using Plot Profile. Numbers of protrusions were counted manually.

### Quantification of PM localization

Co-incidence of Cy3-PI3Kα and GFP–PM marker fluorescence was quantified in reconstructed 3D images of individual cells using Imaris v7.4 software (Bitplane Scientific Software) by measuring how much red immunofluorescence (Cy3-PI3Kα) overlaps with green (GFP–PM marker). 3D image files were cropped to a single cell, then surfaces were created for the red and green channels using settings of Smooth=0.5 μm and Background subtraction=2.5 μm. The green surface was masked and sum of the voxel intensities of the red immunofluorescence inside the mask (i.e. that overlaps with green surface) was calculated as a percentage of the sum of the voxel intensities of all red immunofluorescence to give the percentage of Cy3-PI3Kα that is co-localized with the PM marker. For individual cells in time course experiments, the ratio of the percentage of Cy3-PI3Kα that is co-localized with the PM marker at each time point compared with the percentage of Cy3-PI3Kα that is co-localized with the PM marker at time zero was calculated.

### Lipid overlay assay

Lipid overlay assays were performed according to the method of Dowler [35]. Lipids were reconstituted and diluted in methanol:chloroform:water (2:1:0.8) and 1 μl aliquots were spotted onto Hybond C Extra nitrocellulose membrane (GE Healthcare) at concentrations of between 4 and 500 pmol/μl. Purified recombinant proteins were diluted in PBS before spotting onto Hybond C. Dried membranes were blocked in blocking buffer [50 mM Tris (pH 7.5) containing 150 mM NaCl, 0.1% (v/v) Tween 20 and 2 mg/ml BSA] for 1 h, then incubated with purified recombinant wild-type or mutant PI3Kα or GST-fusion proteins (1 nM in blocking buffer). Membranes were washed in TBS containing 0.1% (v/v) Tween 20, then bound proteins were detected using p85α or GST antibodies, HRP-conjugated secondary antibodies and chemiluminescence as described above.

### Liposome co-sedimentation assays

Liposome sedimentation assays were carried out essentially as described [36]. Lipid mixtures were dried under nitrogen then resuspended in liposome buffer (25 mM HEPES pH 7.5, 100 mM NaCl, 0.5 mM EDTA) by sonication for 10 min to form liposomes. Purified recombinant wild-type or mutant PI3Kα or GST-fusion proteins were diluted in liposome buffer containing 2 mg/ml BSA and pre-centrifuged at 300000 ***g*** for 20 min at 4°C to remove protein aggregates. Clarified proteins (0.5 μg) and sonicated liposomes (50 μg) were mixed and incubated in liposome buffer containing 1 mg/ml casein, to reduce non-specific binding, for 30 min at room temperature with gentle agitation. Liposome bound proteins were separated from unbound by centrifugation at 17000 ***g*** for 30 min at 4°C then the liposome pellet was washed once with 0.5 ml liposome buffer and recentrifuged at 17000 ***g*** for 30 min at 4°C. 10% of the supernatant and 10% of the pellet were separated by SDS–PAGE and the presence of PI3Kα or GST-Grp1-PH in the supernatant or pellet fractions was detected by immunoblotting using p85α and GST antibodies.

### Affinity precipitations

Purified, recombinant wild-type or mutant p110αEE/p85α complexes (100–3200 ng) and GST fusion proteins (1 μg) were incubated in TBS containing 10% (v/v) FBS to reduce non-specific binding and 25 μl of a 50% slurry of glutathione agarose for 1 h at 4°C with gentle agitation. Beads and bound proteins were pelleted by low-speed centrifugation for 10 s and the supernatant removed. Pelleted beads were washed three times with 1 ml ice-cold TBS containing 0.1% Tween 20 and bound proteins were eluted using SDS–PAGE sample buffer. Bound PI3Kα was detected by immunoblotting using p85α antibodies.

## RESULTS

### Fluorescently labelled wild-type and mutant PI3Kα are active *in vitro* and *in vivo*

Complexes of full-length recombinant human p110α and p85α, the prototypic form of Class 1A PI3K, were expressed and purified essentially as described previously for bovine PI3Kα [32] using the strategy of selecting for the p110α/p85α complex by placing a 6 amino acid epitope tag, known as the glu- or EE-tag [37], at the C-terminus of the p110α, the subunit with limiting expression levels [30], which allowed the purification of an enzyme complex with a 1:1 ratio of catalytic:regulatory subunits. The addition of the C-terminal EE tag to p110α did not affect PI3K, as recombinant p110αEE/p85α had high stability and the expected activity and substrate specificity [30].

**Figure 1:**
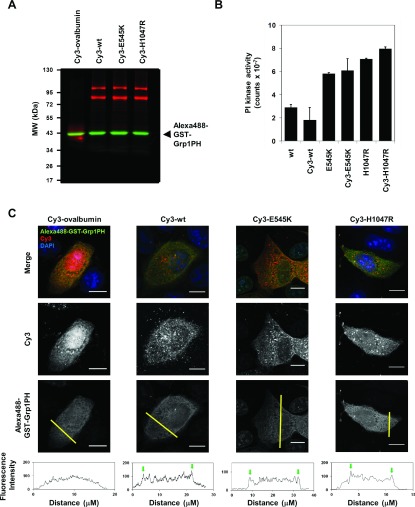
Fluorescently labelled PI3Kα retains lipid kinase activity *in vitro* and *in vivo* (**A**) 2 μg each Cy3-labelled ovalbumin (Cy3-ovalbumin) and Cy3-labelled PI3Kα (Cy3-p110αEE^WT^/p85α, Cy3-p110αEE^E545K^/p85α and Cy3-p110αEE^H1047R^/p85α) were mixed with 2 μg Alexa488-labelled GST-Grp1PH (Alexa488-GST-Grp1PH) then separated by SDS–PAGE. The fixed, unstained gel was visualized using a fluorescence gel scanner with a 532 nm laser and filters for Alexa488 (green) and Cy3 (red). (**B**) 200 ng purified, recombinant Cy3-labelled or unlabelled p110αEE^WT^/p85α, p110αEE^E545K^/p85α or p110αEE^H1047R^/p85α were assayed for phosphoinositide kinase activity. Reactions were stopped after 40 min using 1 M HCl. The difference in phosphoinositide kinase activity between each unlabelled and Cy-labelled PI3Kα was not significant (p>0.1). (**C**) 0.5 μM Cy3-ovalbumin, Cy3-p110αEE^WT^/p85α, Cy3-p110αEE^E545K^/p85α or Cy3-p110αEE^H1047R^/p85α were mixed with 0.5 μM Alexa488-Grp1PH and co-microinjected into adherent MDCK cells in DMEM containing 10% FBS. Cells were allowed to recover for 2 h post-microinjection, then fixed and stained with the nuclear stain, DAPI, and fluorescence associated with Cy3 (middle panel) or Alexa488 (lower panel) was visualized using confocal microscopy. Merged images of Z series projections of Cy3 (red), Alexa488 (green) and DAPI (blue) are shown (top panel). Graphs of Alexa488 fluorescence intensity along the yellow line (lower panel) demonstrate accumulation of Alexa488-GST-Grp1PH at the boundaries of the cell (green arrows). Scale bar=20 μm.

High purity wild-type or mutant p110αEE/p85α complexes (p110αEE^WT^/p85α, p110αEE^E545K^/p85α and p110αEE^H1047R^/p85α) were covalently labelled *in vitro* with the fluorophore Cy3 (Figure 1A) as previously described [32]. Between 2 and 4 Cy3 molecules were incorporated per PI3Kα heterodimer. To ensure that covalent labelling of PI3Kα did not disrupt its structure, wild-type and mutant Cy3-PI3Kα complexes were assayed for their phosphoinositide kinase activity *in vitro*, which were found to be not significantly different to that for unlabelled PI3Kα (Figure 1B). The *in vitro* phosphoinositide kinase activities of the oncogenic mutants, p110αEE^E545K^/p85α and p110αEE^H1047R^/p85α, were higher than that for p110αEE^WT^/p85α (Figure 1B), as previously reported [30,38,39].

Fluorescently labelled wild-type and mutant Cy3-PI3Kα (Cy3-p110αEE^WT^/p85α, Cy3-p110αEE^E545K^/p85α and Cy3-p110αEE^H1047R^/p85α) were introduced into live cells by microinjection [34]. 0.5–2 μM Cy3-PI3Kα was microinjected into each cell, which equates to 3000–100000 molecules of Cy3-PI3Kα per cell assuming that 1–10% of the cell volume (1–2 pl for MDCK cells, [40]) was injected. To ensure that microinjected, fluorescently labelled wild-type and mutant PI3Kα retained enzymatic activity, production of PIP_3_ at the PM was measured using the PH domain from Grp1, a PIP_3_ sensor [41,42]. A purified recombinant fusion protein of GST and the PH domain from Grp1 (GST–Grp1PH) was covalently labelled *in vitro* with Alexa488 (Figure 1A). Equal amounts of Alexa488–GST–Grp1PH and Cy3-PI3Kα (or Cy3-ovalbumin as a control) were mixed (Figure 1A) and co-microinjected into MDCK cells in media containing 10% FBS (Figure 1C). Cells were incubated in the same media for 2 h post-microinjection then fixed and co-stained with DAPI to visualize the cell nuclei. Co-microinjection of Alexa488–GST–Grp1PH with a Cy3-labelled control, Cy3-ovalbumin (Figure 1C), did not lead to detectable recruitment of Alexa488–GST–Grp1PH to the PM. Co-microinjection of Alexa488–GST–Grp1PH with Cy3-p110αEE^WT^/p85α, Cy3-p110αEE^E545K^/p85α and Cy3-p110αEE^H1047R^/p85α resulted in a small but detectable accumulation of Alexa488–GST–Grp1PH at the PM, which was detected as increased Alexa488 fluorescence at the edge of a cross sectional profile of the cell (Figure 1C). These data demonstrate that microinjection of fluorescently labelled forms of PI3Kα leads to the production of low levels of PIP_3_ at the PM of cells maintained in the growth medium, which indicates that Cy3-labelled recombinant PI3Kα remains active when microinjected into adherent cells.

Although the *in vitro* phosphoinositide kinase activities of p110αEE^E545K^/p85α and p110αEE^H1047R^/p85α were higher than that for p110αEE^WT^/p85α (Figure 1B), the amount of Alexa488–GST–Grp1PH, and therefore PIP_3_, at the PM was not measurably greater in p110αEE^E545K^/p85α and p110αEE^H1047R^/p85α-injected cells compared with p110αEE^WT^/p85α-injected cells. Overexpression of mutant PI3Kα has been shown to result in increased levels of pAkt [9,10,14] downstream of PIP_3_; however, lower levels of mutant p110α expression are correlated with lower pAkt [43]. The level of Alexa488–GST–Grp1PH, and therefore PIP_3_, at the PM was very low in PI3Kα-microinjected cells, suggesting that the amount of PI3Kα injected was also very low. An advantage of microinjection is that the amount of protein injected per cell is consistent between cells and between experiments and can be kept low relative to overexpression by transfection of cDNAs, which may better reflect the reported low levels of endogenous p110α and p85α [44]. Low levels of micro-injected PI3Kα may therefore result very small differences in the low levels of PM-associated Alexa488–GST–Grp1PH between cells injected with wild-type and mutant PI3Kα.

### Oncogenic mutant PI3Kα does not associate with PM lipids

Because the lipid substrate for PI3Kα, PIP_2_, is located on the inner leaflet of the PM, recruitment of cytoplasmic PI3Kα to the PM through its binding to PM localized proteins, such as activated RTKs or activated Ras, is an important step in the activation of its lipid kinase activity [1,45]. It has been proposed that the increased activity of the oncogenic H1047R mutant is at least partly due to increased direct binding to PM lipids [25–27,46] and therefore greater access to its substrate. To further test this idea, we measured the association of p110αEE^WT^/p85α, p110αEE^E545K^/p85α and p110αEE^H1047R^/p85α with membrane lipids *in vitro* and in live cells.

**Figure 2:**
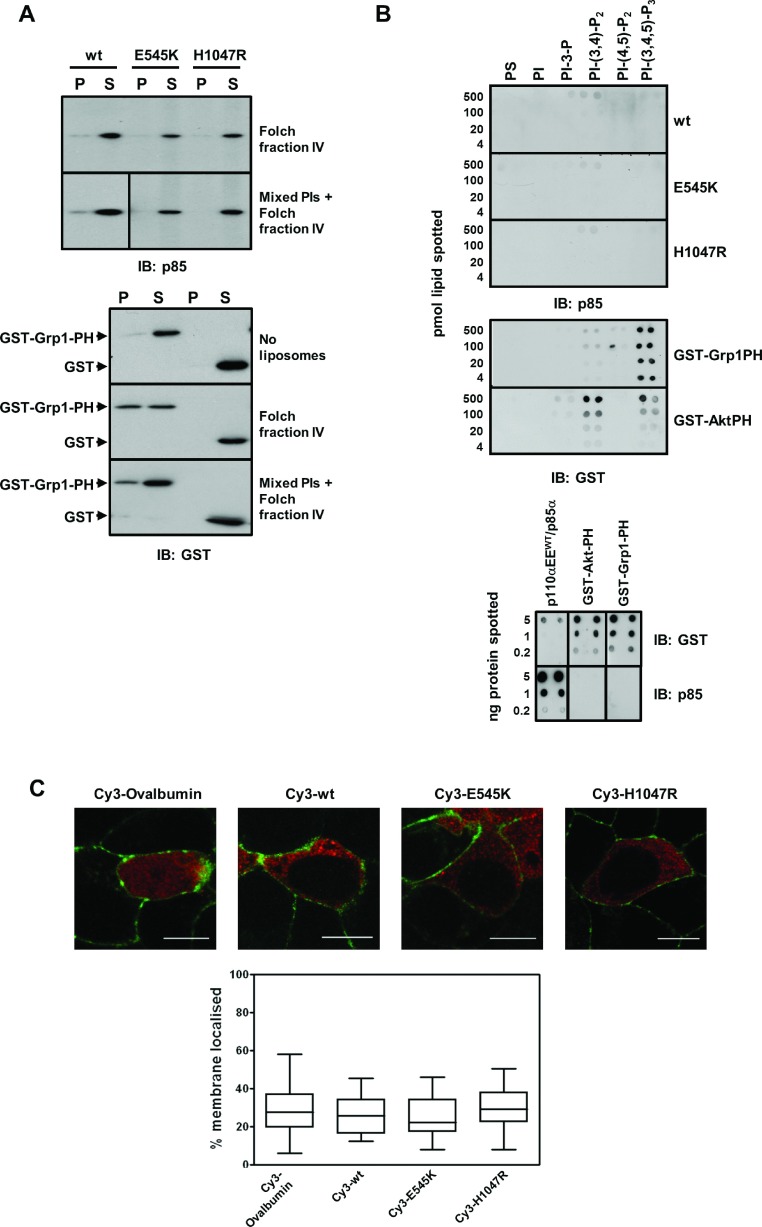
Mutant or wild-type PI3Kα does not bind PM lipids *in vitro* or *in vivo* (**A**) Liposomes derived from 25 μg/ml brain-derived cellular lipids (Folch fraction IV) or 12.5 μg/ml brain-derived cellular lipids plus 12.5 μg/ml mixed PIs were incubated with 5 μg/ml wild-type or mutant (E545K or H1047R) PI3Kα (upper panel) or 5 μg/ml GST-Grp1PH or GST (lower panel). Liposomes were pelleted by centrifugation and liposome-bound protein in 10% of the pellet fraction (P) and free/unbound protein in 10% of the supernatant fraction (S) was detected by immunoblotting using antibodies that recognize p85α or GST. (**B**) 4–500 pmol purified PS or a phosphoinositide was spotted onto a nitrocellulose membrane. Membranes were blocked then incubated with 10 nM wild-type or mutant (E545K or H1047R) PI3Kα (upper panel) or 10 nM GST-Grp1PH or GST-Akt PH (middle panel). Bound PI3Kα, GST-Grp1PH or GST-AktPH was detected by immunoblotting using antibodies that recognize p85α or GST. 0.2–5 ng PI3Kα or GST fusion proteins spotted directly onto nitrocellulose and detected using p85α or GST antibodies (lower panel) were used as positive controls for immunoblotting. (**C**) 0.5–2 μM Cy3-ovalbumin, Cy3-p110αEE^WT^/p85α, Cy3-p110αEE^E545K^/p85α or Cy3-p110αEE^H1047R^/p85α was microinjected into adherent MDCK cells expressing PM-localized GFP in DMEM containing 10% FBS. Cells were allowed to recover for 2 h post-microinjection, then fixed and washed in PBS. Fluorescence associated with Cy3 (red) or GFP (green) was visualized using confocal microscopy (upper panel) (scale bar=10 μm). The percentage of Cy3-PI3Kα fluorescence that was co-incident with GFP-PM marker fluorescence was quantified in 80 successive focal planes in individual cells using Imaris v7.4 software. The median, 25th and 75th percentiles of the percentage of Cy3-PI3Kα co-incident with the PM were calculated from measurements of a minimum of 25 individual cells from three independent experiments and plotted as a box-and-whiskers plot.

The interaction of purified, recombinant wild-type and mutant PI3Kα with liposomes composed of cellular lipids was measured using a liposome co-sedimentation assay (Figure 2A) under conditions in which PI3Kα was capable of phosphorylating liposomes as shown in Figure 1(B). The specificity of the assay was confirmed using GST–Grp1PH and GST alone as a control. GST did not interact with liposomes composed of either brain-derived cellular lipids (Folch fraction IV) or brain-derived cellular lipids supplemented with extra mixed phosphoinositides (mixed PIs+Folch fraction IV). GST–Grp1PH co–sedimented with both types of liposomes but did not aggregate or sediment on its own as it was not found in the pellet fraction in the absence of liposomes. In comparison, PI3Kα did not co-sediment with either brain-derived lipid liposomes or liposomes containing phosphoinositides to a significant extent. There was no evidence for increased association of liposomes composed of cellular lipids with p110αEE^E545K^/p85α or p110αEE^H1047R^/p85α compared with p110αEE^WT^/p85α.

Because it has been proposed that H1047R interacts with membrane lipids via its C2 domain [47] and C2 domains from a number of proteins have been shown to specifically bind phosphoinositides [48–50], the interaction of purified, recombinant wild-type and mutant PI3Kα with phosphoinositides was measured using a Protein Lipid Overlay assay [35] (Figure 2B) with detection of phosphoinositide-bound proteins by p85α or GST antibodies, both of which could detect 1ng of PI3Kα or GST fusion protein that had been directly spotted on to the membrane (Figure 2B, lower panel). Purified GST fusion proteins of the PH domains from Grp1 and Akt1 (GST–Grp1PH and GST–AktPH) specifically bound to PIP_3_ and PIP_3_ plus PI-(3,4)-P_2_ respectively as expected [51]. However, there was little detectable interaction of either wild-type or oncogenic mutant PI3Kα with any of the purified phosphorylated phosphoinositides or with PS. A low-level signal for PI3Kα binding to PI-(3,4)-P_2_ was detectable at longer exposures (Supplementary Figure S1 available at http://www.bioscirep.org/bsr/034/bsr034e104add.htm), but even after a 30 min exposure, there was no evidence for increased binding of p110αEE^E545K^/p85α or p110αEE^H1047R^/p85α to phosphoinositides compared with p110αEE^WT^/p85α.

Next, the amount of microinjected Cy3-PI3Kα located at the PM was measured in cells maintained in growth medium (Figure 2C). Confluent MDCK cells, that had been infected with a construct that leads to expression of PM-targeted GFP, were microinjected with Cy3-labelled PI3Kα (or Cy3-ovalbumin as a control), incubated in media containing 10% FBS for 2–4 h then fixed. Unmounted cells were imaged using confocal microscopy (Supplementary Figure S2 available at http://www.bioscirep.org/bsr/034/bsr034e104add.htm), and 80 Z sections were collected. The amount of Cy3-derived fluorescence that was co-incident with GFP-derived fluorescence was quantified in 3D using Imaris software. The Cy3-labelled control protein, Cy3-ovalbumin, would not be expected to specifically localize to the PM, thus the percentage of Cy3-ovalbumin that is coincident with PM-localized GFP (PM-GFP) provides a measure of the background co-incident fluorescence. The percentage of Cy3-p110αEE^WT^/p85α, Cy3-p110αEE^E545K^/p85α and Cy3-p110αEE^H1047R^/p85α that was coincident with PM-GFP was not significantly greater than that for Cy3-ovalbumin, indicating that the majority of PI3Kα is cytoplasmic in cells cultured continuously in media containing 10% FBS. Importantly, the proportion of p110αEE^E545K^/p85α or p110αEE^H1047R^/p85α localized at the PM was not significantly higher than that of p110αEE^WT^/p85α, indicating that oncogenic mutant PI3Kα is not constitutively localized at the PM in MDCK cells, and therefore suggesting that increased phosphorylation of PIP_2_ and Akt in cells expressing oncogenic mutant PI3Kα is not directly attributable to increased substrate access due to membrane lipid binding.

**Figure 3:**
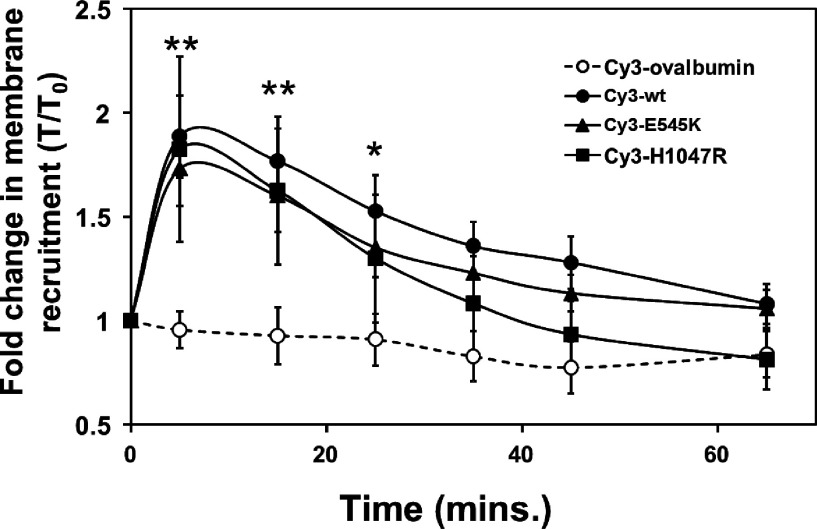
Mutant PI3Kα is not localized to the PM to a greater extent or for an extended time upon cell stimulation compared with wild-type PI3Kα 0.5–2 μM Cy3-ovalbumin (○), Cy3-p110αEE^WT^/p85α (●), Cy3-p110αEE^E545K^/p85α (▲) or Cy3-p110αEE^H1047R^/p85α (■) was microinjected into adherent MDCK cells expressing membrane-localized GFP in DMEM containing 10% FBS. Cells were allowed to recover for 2 h post-microinjection, then starved for 4 h and imaged prior to stimulation and at 5, 15, 25, 35, 45 and 65 min post-stimulation with 10% FBS, 50 ng/ml EGF and 5 μg/ml insulin. The percentage of Cy3-PI3Kα fluorescence that was coincident with GFP-PM marker fluorescence was quantified in 25 successive focal planes in individual cells at each time point using Imaris v7.4 software and expressed as the ratio of the percentage of Cy3-PI3Kα that is co-localized with the PM marker at each time point compared with the percentage of Cy3-PI3Kα that is co-localized with the PM marker at time zero (T/T_0_). The means±S.E.M. of the T/T_0_ ratio was calculated from measurements of a minimum of five individual cells from three independent experiments at each time point. **=*P*<0.05 *=*P*<0.1 compared with time 0.

It has been reported that oncogenic mutant PI3K must be activated by pY binding to the nSH2 domain of p85α before increased binding of mutant PI3Kα to PM lipids *in vitro* is apparent [27]. To test whether greater amounts of mutant Cy3-PI3Kα are recruited to the PM or whether mutant Cy3-PI3Kα is recruited to the PM for a longer period of time upon RTK activation in stimulated cells, the percentage of Cy3-p110αEE^WT^/p85α, Cy3-p110αEE^E545K^/p85α or Cy3-p110αEE^H1047R^/p85α that was co-incident with PM-GFP was measured in the live cells that had been starved for 4 h and at a number of time points post-stimulation (Figure 3). Stimulation of MDCK cells resulted in production of PIP_3_ at the PM, as evidenced by the recruitment of a GFP-PH domain reporter to the PM (Supplementary Figure S3 available at http://www.bioscirep.org/bsr/034/bsr034e104add.htm). Cy3-ovalbumin was not recruited to the PM to a significant extent over a 65 min time-course post-stimulation, confirming that the microinjected Cy3-ovalbumin control remains cytoplasmic as expected. The background level of the coincident Cy3-ovalbumin and PM-GFP fluorescence was the same as in fixed cells (Figure 2C). The level of coincident Cy3 and PM-GFP fluorescence in Cy3-PI3Kα-microinjected cells in starved, unstimulated cells was also the same as for the Cy3-ovalbumin control, indicating that Cy3-PI3Kα is predominantly cytoplasmic both in cells maintained in the growth medium containing 10% FBS (Figure 2C) and in serum-starved cells (Figure 3). Membrane recruitment of microinjected Cy3-PI3Kα was clearly detected using this assay. Upon stimulation, the level of co-incident Cy3-p110αEE^WT^/p85α and PM-GFP increased, peaking at a between 1.5- and 2-fold increase in the percentage of Cy3-PI3Kα that is co-localized with the PM marker at approximately 5 min post-stimulation and declining to near baseline levels after approximately 40 min (Figure 3), which correlates well with the known timecourse for appearance of pAkt post-stimulation [52]. Similarly, the levels of Cy3-p110αEE^E545K^/p85α and Cy3-p110αEE^H1047R^/p85α fluorescence co-incident with PM-GFP fluorescence increased upon stimulation. Although only a very small proportion of total cytoplasmic Cy3-p110αEE^WT^/p85α was recruited to the PM at 5 min post-stimulation (Supplementary Figure S4 available at http://www.bioscirep.org/bsr/034/bsr034e104add.htm), the maximum levels of Cy3-p110αEE^E545K^/p85α and Cy3-p110αEE^H1047R^/p85α recruited to the PM were not significantly greater than that of Cy3-p110αEE^WT^/p85α. In addition, a time course showed that recruitment of p110αEE^E545K^/p85α or Cy3-p110αEE^H1047R^/p85α to the PM was not significantly extended temporally compared with that of Cy3-p110αEE^WT^/p85α. This suggests that increased PI3Kα activity in cells expressing oncogenic mutant PI3Kα is not a direct result of increased substrate access associated with increased membrane lipid binding and concomitant increased localization at the PM in either unstimulated cells or upon RTK activation.

### Microinjection of PI3Kα containing the E545K oncogenic mutation leads to increased numbers of cell protrusions

**Figure 4:**
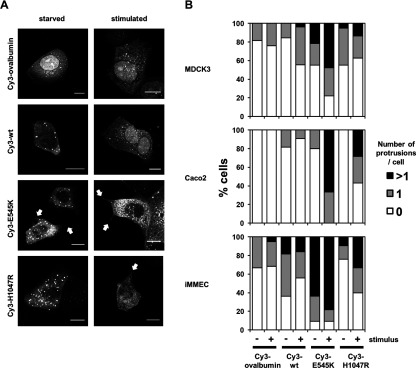
The E545K oncogenic mutation is associated with increased number of cells with cell membrane protrusions (**A**) 0.5–2 μM Cy3-ovalbumin, Cy3-p110αEE^WT^/p85α, Cy3-p110αEE^E545K^/p85α or Cy3-p110αEE^H1047R^/p85α were microinjected into adherent MDCK cells in DMEM containing 10% FBS. Cells were allowed to recover for 2 h post-microinjection, then starved for 4 h before stimulation with 10% FBS, 50 ng/ml EGF and 5 μg/ml insulin for 10 min. Unstimulated and stimulated cells were fixed, and the fluorescence associated with Cy3 was visualized using confocal microscopy. Merged images of Z series projections are shown. Arrows indicate protrusions. Scale bar=10 μm (**B**) 0.5–2 μM Cy3-ovalbumin, Cy3-p110αEE^WT^/p85α, Cy3-p110αEE^E545K^/p85α or Cy3-p110αEE^H1047R^/p85α were microinjected into adherent MDCK, Caco2 or iMMEC epithelial cells in media containing 10% FBS. Cells were treated as described in Figure 4(A) and the number of cells with either 0, 1 or greater than one protrusion was counted. Between 10 and 100 individual cells from at least two independent experiments were scored for each condition.

During microinjection experiments to assess PM recruitment of wild-type and mutant PI3Kα, it was observed that cells microinjected with p110αEE^E545K^/p85α exhibited increased numbers of cell protrusions compared with cells injected with either the control Cy3-ovalbumin, p110αEE^WT^/p85α or p110αEE^H1047R^/p85α. This effect was greater in stimulated cells compared with either cells maintained in 10% FBS or starved cells. In confluent MDCK epithelial cells, which form a typical epithelial sheet with a cobblestone morphology [28], microinjection of Cy3-ovalbumin did not lead to an observable change in cell shape (Figure 4A). In addition, Cy3-ovalbumin was not observed to be recruited to the PM in stimulated MDCK cells, which supports the 3D quantitative data (Figure 3). For microinjected Cy3-p110αEE^WT^/p85α, Cy3-p110αEE^E545K^/p85α and Cy3-p110αEE^H1047R^/p85α, stimulation resulted in a small proportion of cytoplasmic Cy3-labelled protein accumulating at the margins of the cell, which is indicative of recruitment to the PM (Figure 4A). Microinjection of Cy3-p110αEE^WT^/p85α did not result in significant morphology changes to either starved or stimulated MDCK cells. However, microinjection of Cy3-p110αEE^H1047R^/p85α, resulted in a proportion of cells that were more angular than the typical MDCK morphology, and stimulation led to an increased proportion of these angular and asymmetrical cells. Microinjection of Cy3-p110αEE^E545K^/p85α resulted in an increased number of cells with distinct cell protrusions, and stimulation of these cells both increased the proportion of cells with protrusions and the length of the protrusions. In some stimulated cells, Cy3-p110αEE^E545K^/p85α was observed to accumulate at the tips of the cell protrusions.

To simplify quantification of cell morphology, cells were scored for the absence, presence of 1 protrusion or greater than 1 protrusion per microinjected cell (Figure 4B). The number of protrusions varied between cell lines due to their different morphology. MDCK epithelial cells and Caco2 colon epithelial cells form a polarized epithelial monolayer in cell culture, while iMMEC did not form tight cell–cell adhesions and grew as single cells. In addition, cell stimulation increased the proportion of cells with protrusions in all cell types and the morphology of microinjected cells varied depending on whether they were in the centre or at the edge of a cell sheet, thus between 10 and 100 cells were scored for each cell type. Non-microinjected or Cy3-ovalbumin microinjected MDCK and Caco2 cells formed no or few protrusions (Figure 4B), while a number of iMMEC had discernible protrusions associated with their more spiky, single-cell morphology. Microinjection of Cy3-p110αEE^E545K^/p85α resulted in an increased proportion of cells with observable cell protrusions in all three epithelial cell types, and this was exacerbated in stimulated cells. Microinjection of Cy3-p110αEE^WT^/p85α or Cy3-p110αEE^H1047R^/p85α resulted in a small increase in the proportion of cells with protrusions, which was also further increased in stimulated cells; however, the difference between p110αEE^WT^/p85α and Cy3-p110αEE^H1047R^/p85α was marginal and probably not significant. Introduction of p110αEE^E545K^/p85α into live cells is therefore associated with a change in cell morphology and increased numbers of cells with membrane protrusions.

### The E545K oncogenic mutation leads to increased PI3Kα binding to Rho-subfamily small GTPases

**Figure 5:**
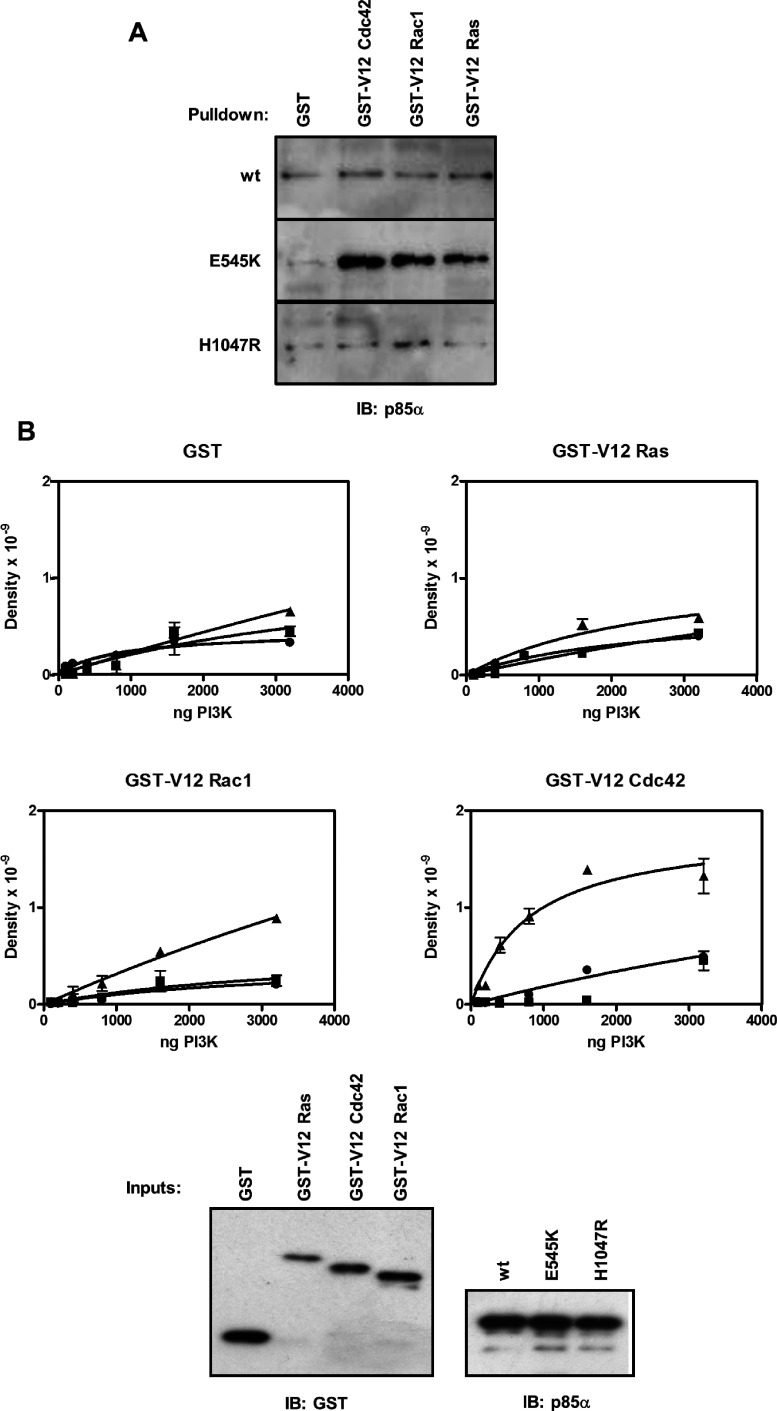
The E545K oncogenic mutated form of PI3Kα specifically binds to constitutively active Cdc42 (**A**) The amount of purified, recombinant wild-type (wt) or oncogenic mutant (E545K or H1047R) PI3Kα bound to 1 μg GST, GST-V12 Cdc42, GST-V12 Rac1 or GST-V12 Ras in the presence of 10% FBS was measured by immunoblotting using an antibody that recognizes p85α. (**B**) 1 μg GST, GST-V12 Cdc42, GST-V12 Rac1 or GST-V12 Ras was incubated with increasing amounts of p110αEE^WT^/p85α (●), p110αEE^E545K^/p85α (▲) or p110αEE^H1047R^/p85α (■). The amount of bound PI3Kα was measured by immunoblotting using an antibody that recognizes p85α and the density of each band was quantified using ImageQuant software. Duplicate points were plotted as means±S.E.M. 1% of the amount of each PI3Kα and GST-fusion protein used in the binding assays (Inputs) was measured by immunoblotting using antibodies that recognize p85α or GST or ensure that equivalent levels of protein were used in each assay.

A number of lines of evidence suggest that at least part of the action of PI3Kα on the actin cytoskeleton that leads to cell membrane protrusions such as lamellopodia and filopodia, cell polarization and ultimately cell migration is mediated through the Ras- and Rho-subfamily GTPases [16,21,53–56]. In addition, the p110α subunit of PI3Kα binds to Ras through the Ras-binding domain [57], and the p85 subunit has been reported to bind the Rho-subfamily GTPases, Rac and Cdc42, through its N-terminal GAP-like or BH (BCR-homology) domain [18,58]. More recently, Rac1 has also been shown to bind to the Ras-binding domain of p110β [59]. To test whether the E545K oncogenic mutation alters interactions with GTPases, which may be implicated in the observed change in morphology of cells microinjected with p110αEE^E545K^/p85α, we measured the interaction of wild-type and mutant purified, recombinant PI3Kα with GST fusion proteins of the constitutively active (V12) forms of human H-Ras, Rac1 and Cdc42Hs. Pull-down assays were carried out in the presence of high protein concentrations (~6–8 mg/ml FBS) to minimize non-specific binding, and under these conditions, the amount of recombinant PI3Kα that bound to GST alone was negligible (Figure 5A). p110αEE^WT^/p85α and Cy3-p110αEE^H1047R^/p85α did not bind to GST-V12 Cdc42, GST-V12 Rac1 or GST-V12 Ras to a greater extent than to GST, while more p110αEE^E545K^/p85α bound to GST-V12 Cdc42, GST–V12 Rac1 and GST-V12 Ras compared with GST (Figure 5A).

To estimate the relative affinities of GST-V12 Cdc42, GST-V12 Rac1 and GST-V12 Ras for p110αEE^E545K^/p85α, the amount of each GST fusion protein bound to increasing concentrations of PI3Kα was measured and the binding isotherms were plotted (Figure 5B). The amounts of GST, GST-V12 Cdc42, GST-V12 Rac1 and GST-V12 Ras used in each assay were similar, as shown. The level of non-specific binding of wild-type and oncogenic PI3Kα to the GST control was similar. Binding of p110αEE^E545K^/p85α to GST-Ras and GST-Rac1 was not saturated at 3200 ng (~250 nM) PI3Kα and therefore these are likely to be relatively low-affinity interactions. In contrast, the binding of p110αEE^E545K^/p85α to GST-Cdc42 was saturable and of a higher affinity compared with that of GST-V12 Rac1 and GST-V12 Ras. The E545K oncogenic mutant therefore specifically binds to constitutively active Cdc42 with appreciable affinity compared with wild-type PI3Kα or the H1047R oncogenic mutant.

## DISCUSSION

Although the subcellular localization and dynamics of PIP_3_ synthesis have been investigated using PIP_3_ sensors that are recruited to the PM when PIP_3_ is present, the subcellular localization of PI3Kα, which produces PIP_3_, has not been well characterized. Currently available antibodies that recognize p110α or p85α do not appear to be of sufficient quality to reliably detect the subcellular location of these proteins by immunofluorescence [23,60]. Expression of PI3Kα by transfection of cDNAs encoding p110α and p85α leads to unequal expression levels of the regulatory and catalytic subunits. The p85α regulatory subunit is generally expressed at high levels relative to p110α and excess free p85α has been shown to act as a dominant negative that inhibits PI3Kα signalling [61,62], whereas the p110α subunit has been shown to be thermodynamically unstable in the absence of p85 [21]. In addition, altering levels of one PI3K subunit alters the relative expression of other subunits [63]. Endogenous PI3K is reported to always be a 1:1 heterodimer of p110 and p85 subunits [64] but the inability to co-express equal levels of p110 and p85 by transient or stable overexpression suggests that ectopically expressed PI3K may not behave like endogenous PI3K.

To test whether increased PM localization is a factor in the higher activity of oncogenic mutant PI3Kα, we chose to take advantage of our ability to express and purify the recombinant p110α/p85α heterodimer in such a way that the ratio of subunits is 1:1 [30]. Highly purified, recombinant p110αEE/p85α was fluorescently labelled *in vitro* with a low molecular mass (*M_W_*=767 Da) CyDye that did not interfere with the enzymatic activity of recombinant PI3Kα (Figure 1B), then introduced into cells by microinjection. This novel technique of pre-forming a protein complex *in vitro* prior to introducing it into cells bypasses the limitations of traditional transfection and overexpression strategies. In addition, microinjection of labelled protein allows imaging of fluorescent proteins within minutes of their introduction into cells, thus minimizing secondary effects due to changes in expression levels of other proteins [34]. The amount of the different PI3Kα complexes and the control protein microinjected was controlled by injecting the same concentrations of recombinant protein under the same conditions, and the amount of protein injected per cell was kept low relative to overexpression by transfection of cDNAs. It was estimated that between 3000 and 50000 molecules of PI3Kα were injected per cell, which is within the range of the reported concentrations of other signalling proteins [40]. Microinjected, fluorescently labelled PI3Kα retained enzymatic activity, as a PIP_3_ sensor was recruited to the PM, albeit at low levels, when PI3Kα, but not a control protein, was microinjected (Figure 1C).

It has been proposed that oncogenic mutants of PI3Kα interact directly with the PM lipid bilayer. The p110 subunit of PI3K contains a C2 domain which, in a number of other proteins, has been shown to bind phosphoinositides [65,66]. However, it is not clear, from the X-ray crystal structure of p110α^H1047R^ in complex with the nSH2 and iSH2 domains of p85α (p85α^nSH2−iSH2^) [25], whether the H1047R mutation alters the accessibility of the putative lipid-binding surface of the C2 domain. Instead, two loops in the kinase domain of p110α^H1047R^ change conformation relative to p110α^WT^ and form a flat, negatively charged surface that was proposed to contact the cell membrane [25]. In addition, the ratio of p110α^H1047R^/p85α^nSH2−iSH2^ to p110α^WT^/p85α^nSH2−iSH2^ PIP_2_ kinase activity was different when PIP_2_-containing liposomes were formed from membrane lipids from different sources, which was interpreted as reflecting the different lipid compositions of the vesicles, suggesting that the H1047R mutation alters the interaction of the kinase domain with the cell membrane.

Even though PI3Kα activity has been shown to be higher in tumour cells containing mutant compared with wild-type PI3Kα [9,10,14], oncogenic mutant forms of PI3Kα were not detected at the PM in cells maintained in the growth medium (Figure 2C). This suggests either that oncogenic forms of PI3Kα do not constitutively interact with the PM or that membrane-associated PI3Kα is below the threshold level of detection in this system. PIP_3_ and pAkt levels in resting or starved tumour cells with oncogenic PI3Kα have been shown to be similar to those in cells with wild-type PI3Kα that have been stimulated with growth factors that activate RTKs [9,10]. PM recruitment of wild-type and mutant PI3Kα was clearly detected in stimulated MDCK cells (Figure 3), suggesting that if the mutant PI3Kα does constitutively bind to PM lipids but cannot be detected in this system, the steady-state levels are much lower than those when wild-type PI3Kα is recruited to the PM post-stimulation. It is not clear whether such a low level of constitutive PM localization could directly result in the same functional outcome, in terms of PIP_3_ and Akt phosphorylation, as does the levels of PM localized PI3Kα detected in stimulated cells (Figure 3).

Interactions of p110α/p85α^nSH2−iSH2−cSH2^ with PIP_2_-containing lipid vesicles have been detected *in vitro* by DXMS (deuterium exchange mass spectrometry) and protein lipid FRET (fluorescence resonance energy transfer) [46]. The interaction of regions of the kinase domain of p110α surrounding the catalytic cleft with membrane vesicles required engagement of the p85α SH2 domains with pY-containing peptides [27,46]. In our system, binding to phosphoinositides or to lipid vesicles was not carried out in the presence of pY-containing peptides in order to test whether there was a significant constitutive interaction between oncogenic mutant PI3Kα and membrane lipids, but no interaction was detected between the wild-type or mutant PI3Kα and membrane lipids *in vitro* and no significant level of PM-localized wild-type or mutant PI3Kα was detected in cells maintained in the growth medium (Figure 2). In growth factor-stimulated cells in which RTKs are activated, PI3Kα was presumably transiently recruited to the PM via interactions with PM-localized pY-containing RTKs or other protein–protein interactions. Only 5–10% of the total PI3Kα was coincident with the PM marker at the peak at 5 min post-stimulation (Supplementary Figure S4), which is similar to the proportion that was shown to co-fractionate with the transmembrane PDGF (platelet-derived growth factor) receptor in PDGF-stimulated cells [67]. However, despite the pY-containing peptide-bound H1047R and E545K oncogenic mutants being shown to bind to lipid vesicles better *in vitro* by DXMS and protein lipid FRET [46], oncogenic mutants of PI3Kα did not persist longer at the PM than wild-type PI3Kα (Figure 3), suggesting that any PI3Kα–lipid interactions that may occur do not outweigh p85αSH2-pY/RTK interactions in determining the amount of time that oncogenic or wild-type PI3Kα is resident at the PM. It is possible that the interaction between the p110α kinase domain and PIP_2_-containing lipid vesicles observed previously [27,46] represents transient enzyme–substrate interactions that would not be sufficiently long-lived to be observed as a change in subcellular localization. However, if this was the case, it would be expected that increased enzyme–substrate affinity leading to increased lipid kinase activity would be reflected in an increased Michaelis constant (*K_m_*) for oncogenic mutant PI3Kα compared with wild-type. The *K_m_* for PIP_2_ has not been measured directly; however, oncogenic mutants have not been reported to have a higher *K_m_* for ATP compared with wild-type PI3Kα but instead display a higher maximum reaction rate (*V_max_*) [30,38,39], suggesting that oncogenic mutant forms of PI3Kα phosphorylate and turn over their lipid substrate more rapidly.

The functional significance of PI3Kα–lipid interactions remains to be determined. While oncogenic mutations increase the intrinsic phosphoinositide kinase activity of PI3Kα by 1.5- to 4-fold [30,38,39], they do not appear to increase its access to its PM-localized substrate (Figure 3). PI3Kα signalling is also tightly regulated by a number of PIP_3_ phosphatases including PTEN (phosphatase and tensin homologue deleted on chromosome 10) and 5-phosphatases [68,69], which are thought to be responsible for the short-lived nature of the PI3Kα product, PIP_3_. Although some lipid phosphatases, notably PTEN, are known as tumour suppressors [69], it is not yet clear whether the magnitude of increase in the intrinsic lipid kinase activity of PI3Kα can override phosphatase regulation to produce the observed increase in Akt signalling without prolonged substrate access.

This raises the possibility that other mechanisms may contribute to the oncogenicity of PI3Kα mutations. p110α and p85α both contain a number of protein–protein interaction domains that can contribute to PI3Kα signalling. In support of this idea, differences between ‘knockout’ and ‘kinase-dead’ mouse models suggest that PI3K may have functions that are independent of its kinase activity [22]. Mutation-specific protein–protein interactions could propagate signalling directly or could conceivably function to localize PI3Kα and PIP_3_ to regions of the cell where PIP_3_ phosphatases are less active. Our observation that microinjection of p110αEE^E545K^/p85α resulted in a morphological change in which an increased proportion of cells had observable protrusions (Figure 4) led us to hypothesize that Rho-subfamily GTPases, such as Rac and Cdc42 [55], could be involved in mediating the effects of the E545K mutation in specific regions of the cell. p110αEE^E545K^/p85α specifically bound to activated Cdc42 (Figure 5), providing some preliminary evidence that this protein–protein interaction may contribute to the oncogenicity of the E545K mutation. Cdc42 is thought to bind to PI3Kα through the p85α GAP-like or BH domain [18,58]. This domain is not present in any of the X-ray crystal structures [18,25,47] or lipid-binding studies [27,46] of PI3Kα but our observation that the E545K mutation is associated with Cdc42 binding suggests that the E545K mutation alters the conformation of PI3Kα to expose the Cdc42-interaction surface on the p85α BH domain. Recently, E545K-mutant PI3Kα was shown to gain the ability to associate with IRS1 [70] but the interaction was between p110α and non-phosphorylated IRS1 rather than the previously reported mechanism of interaction of these proteins, which involved the p85α–SH2 domains and tyrosine phosphorylated IRS1 [71]. It is therefore also possible that Cdc42 interacts with p110α^E545K^, possibly via the Ras-binding domain.

During the initiation of directed cell migration, protrusions form at the leading edge of the cell. Extension of both lamellipodial and filopodial protrusions requires actin polymerization beneath the PM [55], which in the case of filopodia is co-ordinated by Cdc42 [21]. PIP_3_ is found at the leading edge of migrating cells, and PI3K is required for cell polarization [72] and the early steps in EGF- or PDGF-induced cell migration [16]. p110α and Ras have both been shown to be required for EGF-stimulated protrusion formation [16]. Interestingly, Cdc42-regulated filopodia formation has been reported to be dependent on p85α but independent of PI3Kα lipid kinase activity [73]. p110α has also been shown to regulate invadopodia formation [74] and *PIK3CA* mutations are more common in high- versus low-grade colorectal and lung tumours [75] and are associated with increased invasiveness [14]. The finding that p110αEE^E545K^/p85α interacts specifically with constitutively active Cdc42 leads us to speculate that during the initiation of migration or invasion, the activation of both PI3Kα and Cdc42 and their subsequent interaction could be required to propagate a signal that contributes to protrusion formation at the leading edge, and that the E545K mutation may pre-activate PI3Kα, so it is ready to switch on the pathway when Cdc42 becomes activated. Interestingly, *PIK3CA* mutations are associated with K-Ras mutations in advanced tumours [76], which may result in constitutively active Cdc42 in these cells. The p110αEE^E545K^/p85α–Cdc42-binding interface may represent a therapeutic target that could inhibit PI3Kα E545K signalling without affecting wild-type PI3Kα signalling in non-tumour cells.

## Online data

Supplementary data
