# Supplementary material for: Assessing the subcellular distribution of oncogenic phosphoinositide 3-kinase using microinjection into live cells
Source: Biosci Rep. 2014 Apr 14;34(2):e00104. doi: 10.1042/BSR20130133 (PMC3985441; doi:10.1042/BSR20130133)
Supplement: Supplementary data [file bsr034e104add.pdf]

Biosci. Rep. (2014) / **34** / art:e00104 / doi 10.1042/BSR20130133

OPEN ACCESS

Original Paper

Bioscience Reports www.bioscirep.org

## SUPPLEMENTARY DATA

# Assessing the subcellular distribution of oncogenic phosphoinositide 3-kinase using microinjection into live cells

Meredith J. LAYTON*, Natalie K. RYNKIEWICZ*, Ivan IVETAC†, Kristy A. HORAN*, Christina A. MITCHELL* and Wayne A. PHILLIPS*†‡§[1]

*Department of Biochemistry and Molecular Biology, Monash University, VIC 3800, Australia
†Surgical Oncology Research Laboratory, Peter MacCallum Cancer Centre, Melbourne, VIC 3002, Australia
‡Sir Peter MacCallum Department of Oncology, University of Melbourne, Parkville, VIC 3010, Australia
§Department of Surgery (St. Vincent's Hospital), University of Melbourne, Parkville, VIC 3010, Australia

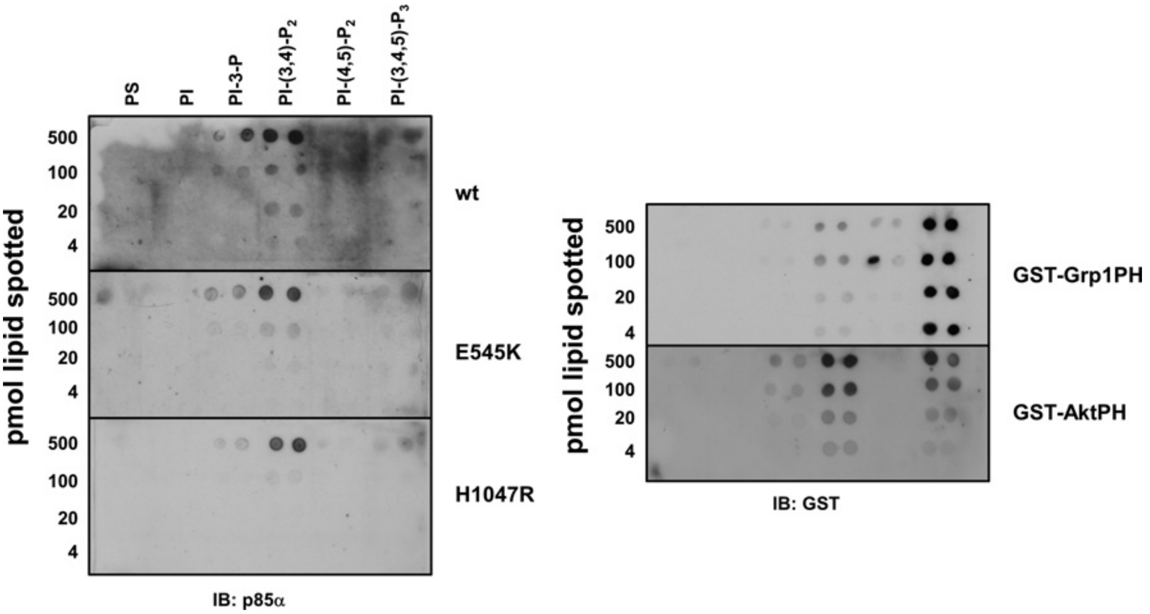


**Figure S1 A longer exposure (30 min) of the lipid overlay assay does not provide evidence for increased binding of mutant PI3Kα to phosphoinositides compared to wild-type PI3Kα**
The nitrocellulose membrane from Figure 2(B) (spotted with 4–500 pmol purified PS or a phosphoinositide) that had been incubated with 10 nM wild-type or mutant (E545K or H1047R) PI3Kα (upper panel) or 10 nM GST-Grp1PH or GST-Akt PH (lower panel) followed by antibodies that recognize p85α or GST was exposed to film for 30 min.

[1] To whom correspondence should be addressed (email wayne.phillips@petermac.org).

© 2014 The Author(s) This is an Open Access article distributed under the terms of the Creative Commons Attribution Licence (CC-BY) (http://creativecommons.org/licenses/by/3.0/) which permits unrestricted use, distribution and reproduction in any medium, provided the original work is properly cited.

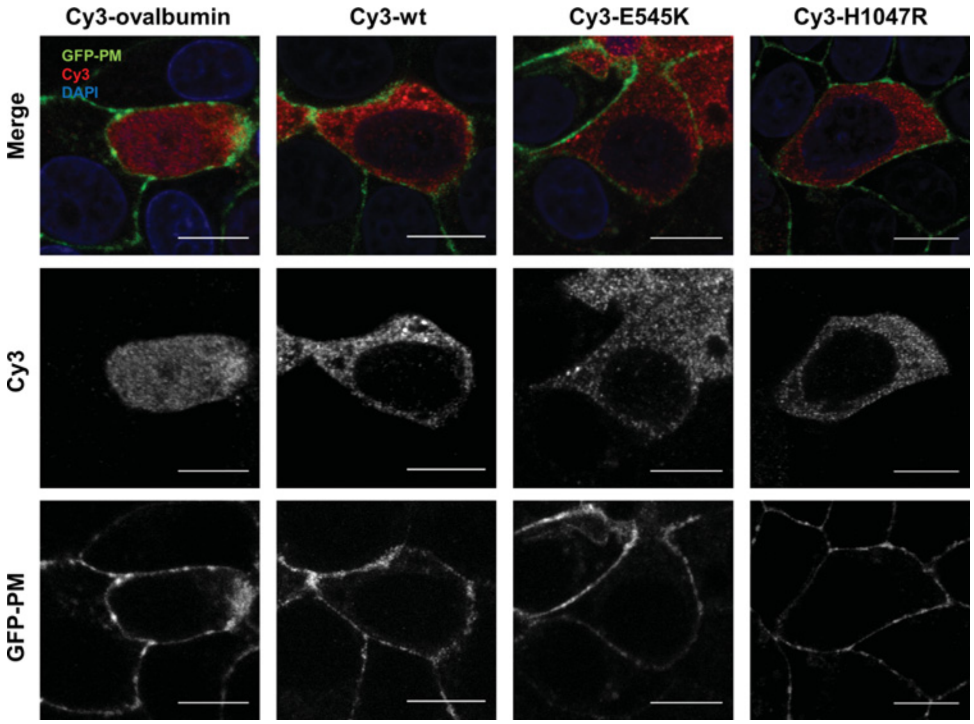


**Figure S2 Single-channel images from Figure 2(C)**

0.5–2 $\mu$M Cy3-ovalbumin, Cy3-p110$\alpha$EE$^{WT}$/p85$\alpha$, Cy3-p110$\alpha$EE$^{E545K}$/p85$\alpha$ or Cy3-p110$\alpha$EE$^{H1047R}$/p85$\alpha$ was microinjected into adherent MDCK cells expressing membrane-localized GFP in DME containing 10% (v/v) FBS. Cells were allowed us to recover for 2 h post-microinjection, then fixed and washed in PBS and stained with DAPI to detect nuclear DNA. Fluorescence associated with Cy3 (red), GFP (green) or DAPI (blue) was visualized using confocal microscopy (upper panel). Single channel images are shown for Cy3 (middle panel) and the GFP-PM (lower panel). Scale bar = 10 $\mu$m.

© 2014 The Author(s) This is an Open Access article distributed under the terms of the Creative Commons Attribution Licence (CC-BY) (http://creativecommons.org/licenses/by/3.0/) which permits unrestricted use, distribution and reproduction in any medium, provided the original work is properly cited.

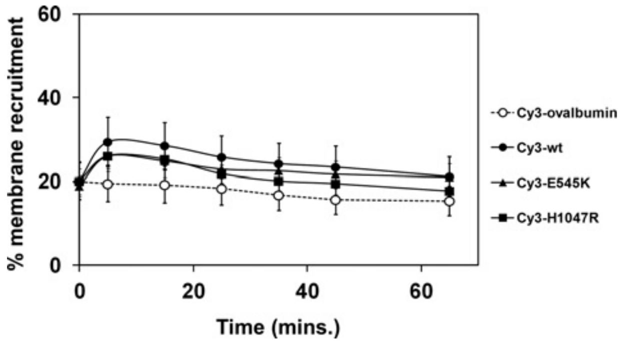


**Figure S3 Data from Figure 3 represented as the means±S.E.M. of the percentage of Cy3-PI3Kα co-incident with the plasma membrane**

0.5–2 μM Cy3-ovalbumin (○), Cy3-p110αEE$^{WT}$/p85α (●), Cy3-p110αEE$^{E545K}$/p85α (▲) or Cy3-p110αEE$^{H1047R}$/p85α (■) was microinjected into adherent MDCK cells expressing membrane-localized GFP in DME containing 10% FBS. Cells were allowed to recover for 2 h post-microinjection, then starved for 4 h and imaged prior to stimulation and at 5, 15, 25, 35, 45 and 65 min post-stimulation with 10% FBS, 50 ng/ml EGF and 5 μg/ml insulin. The percentage of Cy3-PI3Kα fluorescence that was co-incident with GFP-plasma membrane marker fluorescence was quantified in 25 successive focal planes in individual cells at each time point using Imaris v7.4 software. The means±S.E.M. of the percentage of Cy3-PI3Kα co-incident with the plasma membrane were calculated from measurements of a minimum of five individual cells from three independent experiments at each time point.

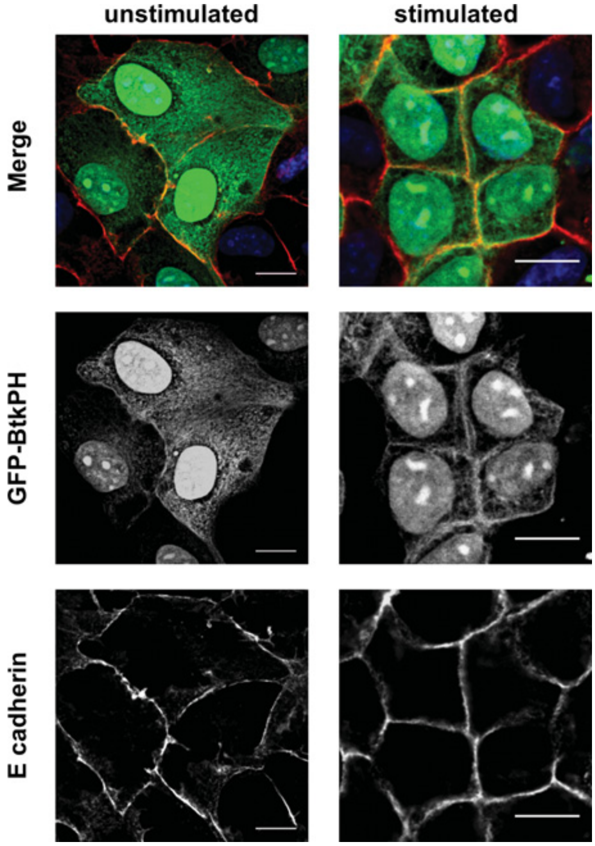


**Figure S4 To measure whether of stimulation of MDCK results in activation of endogenous PI3Kα resulting in $PIP_3$ production, cells were transfected with GFP-BtkPH, a $PIP_3$ reporter [1]**

Two days after transfection, cells were starved for 4 h in media containing no FBS at 37°C then stimulated for 10 min with 10% FBS, 50 ng/ml EGF and 5 μg/ml insulin to activate RTKs. Cells were fixed and washed in PBS and stained with DAPI to detect nuclear DNA and E cadherin, as a PM marker, followed by an Alexa594-labelled secondary antibody (red). Fluorescence associated with GFP (green), Alexa 594 (red) or DAPI (blue) was visualized using confocal microscopy (upper panel). Single-channel images are shown for GFP-BtkPH (middle panel) and E cadherin/Alexa 594 as a PM marker (lower panel). Scale bar = 10 μm.

## REFERENCE

1 Varnai, P., Rother, K. I. and Balla, T. (1999) Phosphatidylinositol 3-kinase-dependent membrane association of the Bruton's tyrosine kinase pleckstrin homology domain visualized in single living cells. J. Biol. Chem. **274**, 10983–10989 CrossRef PubMed

**Received 10 December 2013/18 February 2014; accepted 4 March 2014**

**Published as Immediate Publication 5 March 2014, doi 10.1042/BSR20130133**

© 2014 The Author(s) This is an Open Access article distributed under the terms of the Creative Commons Attribution Licence (CC-BY) (http://creativecommons.org/licenses/by/3.0/) which permits unrestricted use, distribution and reproduction in any medium, provided the original work is properly cited.
